# Amyotrophic lateral sclerosis in Mainland China: clinical translational challenges and opportunities

**DOI:** 10.1097/WCO.0000000000001421

**Published:** 2025-08-20

**Authors:** Ji He, Dongsheng Fan

**Affiliations:** a Department of Neurology, Peking University Third Hospital; b Changping Laboratory, Beijing, China

**Keywords:** amyotrophic lateral sclerosis, biomarkers, Chinese population, clinical trails

## Abstract

**Purpose of review:**

Amyotrophic lateral sclerosis (ALS) imposes a growing medical and socioeconomic burden in China. This review synthesizes recent advances in understanding ALS epidemiology, biomarker discovery, therapeutic innovations, and policy frameworks in China. It highlights the urgency of addressing challenges, including fragmented healthcare resources, translational medicine gaps, and regional inequities, while emphasizing China's unique contributions to global ALS research.

**Recent findings:**

Chinese ALS cohorts exhibit distinct epidemiological profiles, including a younger mean age of onset and prolonged median survival. Policy initiatives, such as ALS inclusion in rare disease registries and insurance reforms, aim to reduce financial burdens of patients. Multimodal biomarker exploration has advanced integrated diagnostic models combining neurofilament light chain (NfL) and clinical data platforms. Neuroimaging and electrophysiological studies reveal glymphatic dysfunction, white matter degeneration, and neuromuscular junction abnormalities, with novel links to hepatic metabolism. Genomic analyses identify population-specific variants. Therapeutic innovations in China include not only biopharmaceuticals, but also integrative traditional Chinese medicine (TCM) approaches.

**Summary:**

China's ALS landscape is transitioning towards precision medicine through biomarker-guided diagnostics and multidisciplinary care models. Key priorities include establishing a national ALS registry, standardizing biomarker validation, and expanding clinical trials to bridge translational medicine gaps.

## INTRODUCTION

Motor neurone diseases (MNDs) represent a heterogeneous group of chronic progressive neurodegenerative disorders characterized by the selective degeneration of anterior horn cells in the spinal cord, brainstem motor neurones, and corticospinal tracts. Amyotrophic lateral sclerosis (ALS), which comprises over 90% of patients with MND [1], is the predominant subtype. Notwithstanding its relatively low global incidence (0.4–2.6 per 100 000 person-years) [2]. ALS aetiopathogenesis remains elusive, and current therapeutic options are limited to disease-modifying agents. The main drugs available to patients with ALS in China include riluzole and edaravone. Riluzole shows modest efficacy in slowing disease progression, while edaravone, a free radical scavenger, mitigates oxidative neuronal damage. In 2024, the Chinese regulatory authorities approved Qalsody to treat patients with *SOD1* mutations [3^▪^].

ALS imposes a growing medical and socioeconomic burden in China due to its large population base and regional healthcare disparities [4]. However, China is poised to develop a proactive ALS clinical management framework with implications for neurodegenerative disease research and treatment. ALS landscape is transitioning towards precision medicine through biomarker-guided diagnostics and multidisciplinary care models. This review synthesizes recent advances in understanding ALS epidemiology, biomarker discovery, therapeutic innovations, and policy frameworks in China. It highlights the urgency of addressing systemic challenges, including fragmented healthcare resources, biomarker translation gaps, and regional inequities, while emphasizing China's unique contributions to global ALS research. 

**Box 1:**
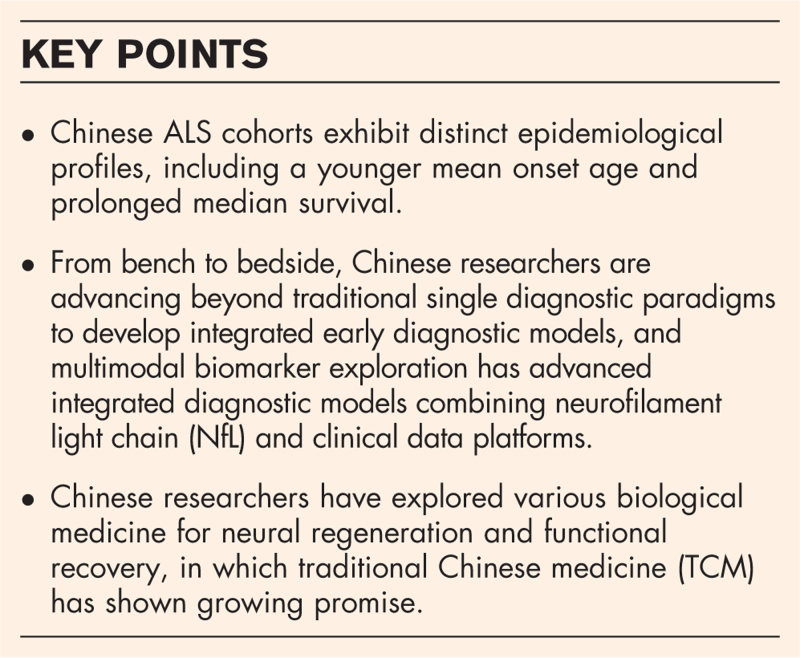
no caption available

## EPIDEMIOLOGICAL AND CLINICAL CHARACTERISTICS IN CHINA

Chinese patients with ALS show distinct demographic patterns than Western populations [5,6]. The mean age of onset was 49.8 years, with limb-onset presentations predominating (similar to Western cohorts); however, bulbar-onset cases did not occur as frequently. The median survival was 71.0 months, surpassing the reported Western averages. In urban populations, the national prevalence and incidence rates were 2.91/100 000 and 1.65/100 000, respectively [5].

The Chinese National Health Commission established the Hospital Quality Monitoring System (HQMS) in 2011 to monitor the performance of public hospitals. All public tertiary hospitals regularly transmit front-page data of inpatient medical records. Analysis of 2023 (released in 2024, Fig. 1) HQMS for patients with ALS data from 1714 tertiary hospitals across mainland China showed a male predominance (64.6%, number of unique hospitalizations: 21 002) over women (35.4%, number of unique hospitalizations:11 529), yielding a 1.8 : 1 sex ratio. The mean age at in-hospital mortality was 70.3 ± 13.7 years. The geographical distribution patterns of patients with ALS in China strongly correlate with regional population density and economic development, reflecting healthcare resource allocation disparities. Patient concentration is higher in areas with large population bases and developed economies.

**FIGURE 1:**
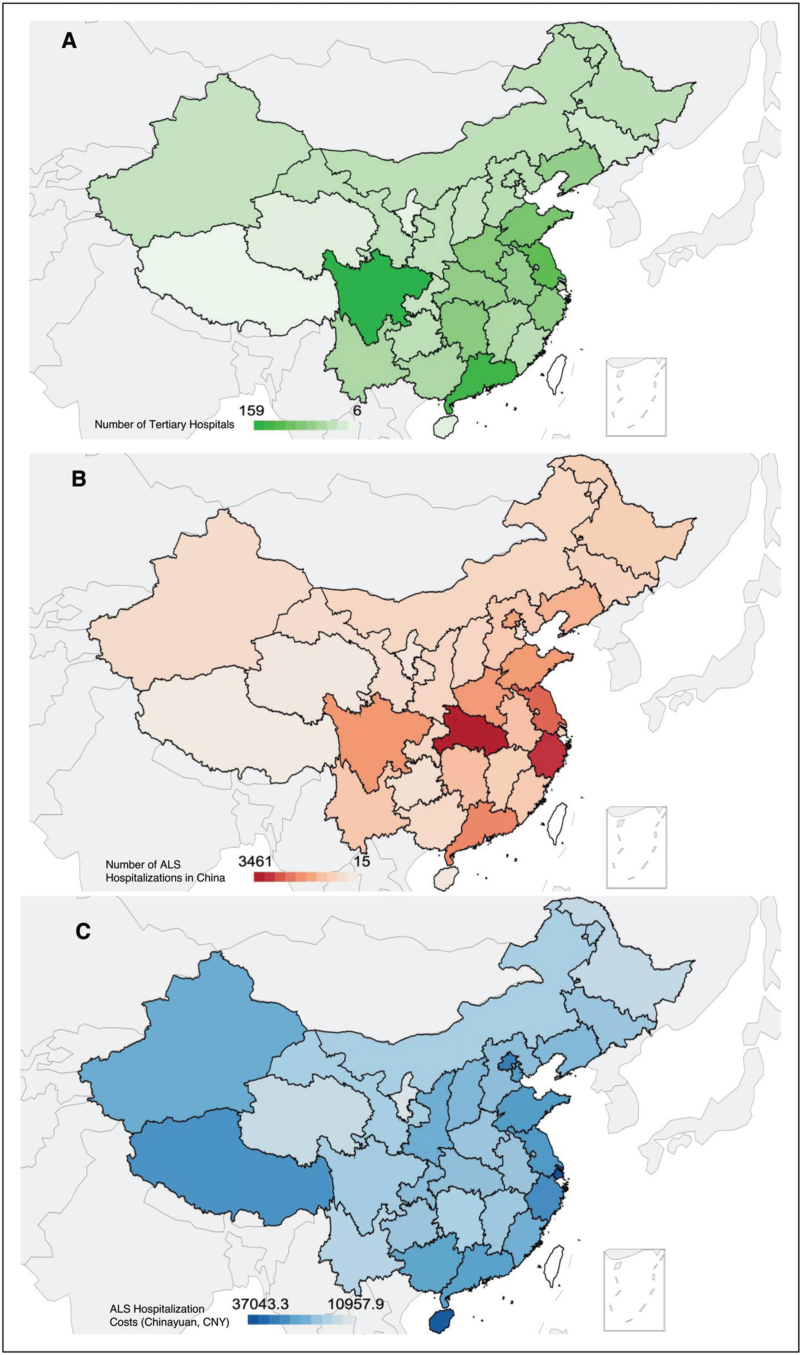
Regional data from rom Hospital Quality Monitoring System (HQMS) on tertiary public hospitals, ALS/MND hospitalisations, and costs in mainland China, 2023. (a) Distribution of 1714 tertiary public hospitals included in the HQMS database across provincial-level regions. (b) Geographic distribution of 32531 ALS/MND hospitalisations reported in tertiary public hospitals. (c) Average total hospitalisation cost per patient by region, highlighting variation in expenditure across provinces.

### Healthcare utilisation and economic burden

The mean hospitalisation cost per ALS admission in tertiary hospitals by 2023 was 22 723.40 (ChinaYuan, CNY), with substantial regional cost variations. Symptomatic management is the primary component of this expenditure. Shanghai shows significantly higher treatment costs than the less-developed regions. These discrepancies highlight both resource allocation inequalities and the potential underestimation of mortality in areas with scarcity of healthcare access, where patients in end-stage often transition to primary care or home hospices.

### Policy initiatives and systemic challenges

China has shown measurable progress in its policy support and social security frameworks for neurodegenerative disorders. ALS inclusion in the *Catalogue of Rare Diseases* announced by the government of China has catalysed the development of standardised diagnostic and treatment systems. Provincial initiatives incorporating ALS into urban-rural resident critical illness insurance and medical assistance programmes have achieved partial cost coverage; nonetheless, nationwide implementation remains incomplete.

China's current ALS healthcare system faces several challenges. Systemic deficiencies in national surveillance data create gaps in epidemiological information that hinder the development of evidence-based management strategies. The economic burden associated with cross regional care seeking, such as travel from rural or smaller cities to urban centers for advanced medical services, not only increases referral, transportation, and accommodation costs but also further strains the already limited capacity of tertiary healthcare institutions [7]. The current symptom-based treatment paradigm offers limited neuroprotective benefits. Although China has participated in international multicentre trials, such as HIMALAYA (ClinicalTrials.gov identifier: NCT05237284), which have accelerated the development of novel therapies, translational bottlenecks persist, with basic research findings failing to translate effectively into clinical practice. As a result, effective interventions that delay or reverse disease progression remain lacking, and patients continue to bear a substantial long-term economic burden. Marked regional disparities are evident, as treatment costs in developed areas, including Shanghai, are three times higher than those in underdeveloped regions, highlighting a pronounced uneven distribution of high-quality medical resources. This requires the establishment of industry-academia-research collaboration mechanisms.

### Multimodal biomarker integration in Chinese amyotrophic lateral sclerosis clinical research

China has established a multidimensional research framework encompassing basic science, clinical translation, and interdisciplinary collaboration in ALS studies. Cohort development has transcended geographical limitations through integration with international biobanks, including the UK Biobank [8]. ALS biomarker investigations focus on biomarker signatures discovery and genetic/environmental risk factors stratification and distinct research priorities ranging from elucidating pathogenic mechanisms and early diagnostic techniques to identifying therapeutic targets and intervention optimisation. Clinical researchers have developed advanced DNA molecular subtyping systems [9], pioneered neuroimaging diagnostics [10], and standardised protocols for managing patients [11]. These innovations inform domestic clinical practice and provide Asian-specific datasets for global ALS etiological studies. Expanding national multicentre research networks and policy frameworks is driving a paradigm shift towards biomarker-guided precision diagnostics and personalised therapeutic regimens with the potential to improve prognostic outcomes.

### Innovations in amyotrophic lateral sclerosis biomarker development

Establishing definitive diagnostic and prognostic biomarkers remains challenging despite recent progress in identifying potential biomarkers for ALS. Chinese researchers are advancing beyond traditional single diagnostic paradigms to develop integrated early diagnostic models, such as bioinformatics strategies [12,13] with TDP-43 protein detection and neurofilament light chain (NfL) quantification. These novel frameworks include genetic information, the dynamic monitoring of metabolic profiles, inflammatory cytokine fluctuations, and ALS functional clinical scores.

Technological innovations include artificial intelligence driven multimodal data integration platforms that process high-dimensional clinical datasets, imaging biomarkers, and molecular profiles [14–16]. They focused on the multidimensional correlations between neuroelectrophysiology, imaging signatures, and molecular biomarker trajectories to improve subclinical detection rates. These systems permit quantitatively modelling disease progression trajectories and therapeutic response evaluation.

### Electrophysiological insights into amyotrophic lateral sclerosis pathogenesis in Chinese patients

From bench to bed, electrophysiological investigations of Chinese ALS cohorts provide insights into disease mechanisms. Advanced techniques like patch-clamp recording, calcium imaging, and microelectrode array analysis have shown characteristic motor neurone dysfunction patterns, anion channel dysregulation, and neural network hyperactivity in patients with *CLCC1* mutations [17]. Recently, the electrophysiological profiling of ALS-derived organoids has been possible, facilitating multiscale analysis from cellular pathophysiology to complex tissue-level network dynamics [18]. In clinical practices, distinctive neurophysiological patterns emerge early in the progression of ALS disease, including a low-frequency decrement in repetitive nerve stimulation indicative of neuromuscular junction dysfunction [19]. Clinically, motor unit number estimation and electrical impedance myography quantitatively evaluate motor neurone loss and muscle degeneration trajectories, improving our understanding of disease progression [20].

### Multimodal imaging insights into amyotrophic lateral sclerosis pathogenesis from Chinese cohorts

Chinese studies have described the pathological imaging features of patients with local ALS characterised by glymphatic dysfunction, white matter microstructural degeneration, and grey matter network remodelling. Glymphatic impairment was determined using the perivascular space index in patients with early-stage ALS, showing significant reductions compared to healthy controls with dynamic correlations to functional rating scale scores and sleep disturbance metrics [21]. Neurite orientation dispersion and density imaging were used in a complementary study to reveal the reduced orientation dispersion index in the thalamic-motor cortical pathways, which correlated with the severity of ALS [22]. A robust inverse correlation between internal capsule fractional anisotropy and cerebrospinal fluid NfL levels was identified [23], mechanistically linking white matter integrity loss to axonal degeneration biomarkers. Notably, Zhu *et al.* [24^▪^] investigated the liver-brain axis interactions revealed hepatic metabolic parameters as independent risk factors for ALS progression, a paradigm shift from traditional central nervous system (CNS)-centric models.

### Advancing amyotrophic lateral sclerosis classification through blood biomarker discovery

Studies on blood biomarkers drive a paradigm shift for diagnosing ALS from a “symptom-driven” to a “molecular subtyping” approach. Widely investigated biomarkers are primarily in cerebrospinal fluid, blood, and urine. Neurofilament proteins [25] and glial fibrillary acidic protein (GFAP) accurately show pathological changes in the CNS; however, creatinine and creatine kinase primarily show pathological alterations in peripheral nerves and muscles [26,27]. The neurofilament light chain remains stable as a marker of neuronal axonal injury throughout disease progression and is a promising diagnostic and prognostic biomarker due to its high specificity and sensitivity, notwithstanding the current challenges in differentiating ALS from other CNS disorders related to axonal injury. GFAP shows the degree of neuronal demyelination and is closely related to nonmotor symptoms of ALS, including cognitive impairment, variations in oxygen saturation, and abnormalities in the glomerular filtration rate [28]. A pathological protein associated with ALS, TAR DNA-binding protein 43 (TDP-43), has emerged as a promising biomarker, especially with advances in exosome-related research [29]. Evidence to support the use of creatinine and creatine kinase as diagnostic markers is currently insufficient; however, they have shown potential for predicting disease prognosis. Advancements in blood biomarker research have improved early diagnosis and precise disease monitoring in ALS. These advancements have provided new insights into its pathological mechanisms and targeted therapies development. Moreover, applying biomarker panels, including chitinases and other clinical indices, overcomes single marker limitations and offers a potential solution for early ALS screening [30].

### Critical challenges in translating amyotrophic lateral sclerosis biomarkers to clinical practice

Current efforts to implement ALS biomarkers may face significant obstacles. First, the high genetic and phenotypic heterogeneity in ALS necessitates multidimensional biomarker panels that integrate genomic, proteomic, and metabolomic profiles, thereby overcoming the limited sensitivity of single-marker approaches. Second, ethnic and sampling biases persist, with most existing data obtained from European and American populations [24^▪^]. These data require correction using biomarker validation studies from non-Caucasian populations. Third, standardisation deficits manifest as inconsistent multicentre validation protocols and the absence of an international consensus on analytical thresholds, especially for novel and potential ALS biomarkers.

### Advancing amyotrophic lateral sclerosis precision medicine through genomic and multiomic integration

China's ALS research initiative involves constructing a large-scale patient genomic database to advance precision medicine by integrating whole-genome sequencing with multiomics profiling. This strategy systematically relates genetic variation patterns with clinical phenotypes like age of onset and disease progression. Chinese patients revealed an important mechanism of ALS pathogenesis. Research in China on patients with sporadic ALS shows a 50.3% prevalence of mitochondrial complex IV dysfunction [31^▪▪^]. This finding suggests an independent pathogenic mechanism. Novel population-specific variants, including *PCDHA9* (p.L700P) [32^▪▪^], have been identified, with corresponding rat models successfully replicating important pathologies, including TDP-43 aggregation and motor neurone loss. These databases show unique genetic features in Chinese ALS [33,34]. Patients with ALS show a high mutation rate in *SOD1*, *FUS*, and *ANXA11*, which are significantly higher than those in Western cohorts characterised by lower frequencies of *C9orf72* mutations. The identification of epigenetic regulation has further shown coordinated neuroinflammatory modulation mediated by dysregulated long noncoding RNAs (lnc-NR3C and lnc-HIBADH-4 [35]) and distinct DNA methylation patterns [36].

Chinese studies also indicate that inflammation is critical in ALS pathogenesis [37,38]. Several study groups have investigated dynamic changes in immune cells and inflammatory mediators, like cytokines and chemokines, during disease progression while exploring the potential of anti-inflammatory or immunomodulatory strategies to delay neuronal degeneration [39]. Recently, the relationship between gut microbiota and ALS has achieved significant attention [40]. The gut microbial composition in patients with ALS was analysed to determine whether dysbiosis contributes to inflammatory responses and neurodegenerative processes and to improve prognosis using gut environment modulation [41,42]. The mechanisms of immune inflammation and the gut-brain axis in ALS are interconnected, forming a complex network mediated by metabolites, immune cells, and neural pathways. Novel therapeutic approaches targeting these intersecting nodes, including RIPK1 inhibitors (even such as mannose related mechanisms [43] ), have advanced to clinical validation stages, while negative results might not be surprising.

### Emerging paradigms in amyotrophic lateral sclerosis management: multidisciplinary integration and innovative therapeutics in China

Managing ALS in China is progressively transitioning towards a multidisciplinary team approach, integrating collaborative efforts across neurology, rehabilitation, psychology, and respiratory medicine to optimise patient care. Chinese research initiatives are building on existing pharmacological interventions like riluzole and edaravone and are exploring drug repurposing strategies to identify novel therapeutic candidates while investigating multitarget combination therapies to disrupt disease progression through complementary mechanisms. Psychological burdens, including anxiety and depression, remain prevalent in patients with ALS and their caregivers. Patient advocacy groups, including the ALS Care Alliance, now provide structured psychosocial support, counselling services, and home care training programmes to address these challenges. Governmental and social organisations offer financial assistance through medical insurance coverage for certain treatments and subsidies for assistive devices.

Antisense oligonucleotide therapy (such as Qalsody) has accumulated significant attention, with advancements in gene therapies that target specific mutations (*SOD1*). Table 1 shows the emerging therapeutic modalities being majorly developed in China, including gene therapy, anti-inflammatory interventions, and stem cell transplantation. Chinese researchers have explored various biological medicine for neural regeneration and functional recovery, while currently mainly offering therapeutic avenues for early-stage patients. Traditional Chinese medicine (TCM) has shown growing promise [44^▪^] in symptomatic management via integrative approaches that synergise the holistic diagnostic paradigms of TCM with modern molecular profiling (Chinese Clinical Trial Registry identifier: ChiCTR2400083114). In China, technological innovations such as brain-computer interface systems are revolutionising the care of patients with end-stage ALS. Electroencephalogram-based communication devices are examples of such breakthroughs [45], being applied into Chinese ALS patients and enabling environmental interactions and significantly improving the patients’ quality of life through enhanced assistive technologies.

**Table 1:** Registered clinical trials for amyotrophic lateral sclerosis therapies majorly developed in China in from 2024 to 2025

No.	Study name	Clinical stage/IIT Phase	Registration number/Implied license number	Currently accessible disclosure
1	A Randomized, Double-Blind, Controlled Clinical Study to Evaluate the Efficacy and Safety of FB1006 in Patients with Amyotrophic Lateral Sclerosis (ALS), Investigator-Initiated.	Investigator-initiated trial	NCT05923905	1. Small molecular medicine (oral medicine) 2. Drug repurposing 3. The study has two phases: the first phase is 24 weeks, using a randomized double-blind placebo-controlled design; the second phase is 24 weeks, using an open-label study design 4. Enrollment number (finished): 64
2	A Multicenter, Single-Arm, Open-Label Exploratory Clinical Study to Evaluate the Safety, Tolerability, and Preliminary Efficacy of a Single Intrathecal Injection of SNUG01 in Patients with Amyotrophic Lateral Sclerosis (ALS).	Investigator-initiated trial	ChiCTR2400090090	1. AAV-medicine 2. Intrathecal injection 3. SNUG01 is a gene therapy based on the self-complementary adeno-associated virus (scAAV) serotype 9. 4. Multicenter study in China
3	A Single-Arm, Open-Label Exploratory Clinical Study to Evaluate the Safety, Tolerability, and Preliminary Efficacy of a Single Intrathecal Injection of SNUG01 in Patients with Amyotrophic Lateral Sclerosis (ALS).	Investigator Initiated Trial	ChiCTR2300076019	1. AAV-medicine 2. Intrathecal injection 3. SNUG01 gene therapy 4. Enrollment number (finished): 7
4	A Multicenter, Randomized, Double-Blind, Placebo-Controlled Clinical Study on the Efficacy and Safety of Xiatongzine in the Treatment of Amyotrophic Lateral Sclerosis (ALS)	Phase II	ChiCTR2000039689	1. TCM (oral medicine) 2. Enrollment number (finished): 155
5	A Multicenter, Randomized, Double-Blind, Positive Drug Parallel-Controlled Clinical Trial to Evaluate the Efficacy and Safety of Huoling Shengji Granules in the Treatment of Amyotrophic Lateral Sclerosis (ALS) with Spleen-Qi Deficiency and Kidney-Yang Deficiency Syndrome	Phase II	ChiCTR2100044085	1. TCM (oral medicine) 2. To evaluate the efficacy and safety of Huolingshengji Keli granules for the treatment of ALS, compared with riluzole. 3. To provide data for NDA or the design of following clinical trials. 4. Enrollment finished
6	A Randomized, Double-Blind, Placebo-Controlled, Dose-Escalation, Multicenter Clinical Study to Evaluate the Efficacy and Safety of Shenrong Granules in the Treatment of Amyotrophic Lateral Sclerosis (ALS) with Du-Yuan Deficiency Syndrome	Phase II	ChiCTR2400083114	1. TCM (oral medicine) 2. To evaluate the effectiveness and optimal effective dose of Shenrong granules in the treatment of ALS (Du yuan deficiency syndrome). 3. Enrollment number: 180
7	A Phase I, Single-Arm, Multicenter, Open-Label Clinical Study Evaluating the Safety and Efficacy of a Single Intrathecal Injection of RJK002 Solution in Patients with Amyotrophic Lateral Sclerosis (ALS)	Phase I	NCT06493279	1. AAV-medicine 2. Intrathecal injection 3. To evaluate the safety and tolerability of a single intrathecal injection of RJK002 in ALS, and to determine the recommended Phase II dose (RP2D). 4. Enrollment number: 9
8	Shize Biotech's Gene-Edited Autologous iPS-Derived Subtype Neural Cell Therapy	Phase I	NCT06765564	Cell Therapy Intrathecal injection Enrollment number: 6
9	A Randomized, Double-Blind, Placebo-Controlled Study Evaluating the Safety, Tolerability, Pharmacokinetics, and Pharmacodynamics of RAG-17 in Patients with Amyotrophic Lateral Sclerosis (ALS) Carrying Superoxide Dismutase 1 (SOD1) Gene Mutations	Phase I	NCT06556394	1. Small-RNA 2. Intrathecal injection 3. The dose levels will be evaluated sequentially across separate cohorts using a rules-based design, wherein participants will receive RAG-17 or placebo. 4. Enrollment number: 32
10	A Phase I Clinical Study Evaluating the Safety, Tolerability, and Pharmacokinetic Characteristics of Single and Multiple Doses of FHND1002 Granules in Healthy Adult Volunteers	Phase I	NCT06782958	1. Small molecular medicine (Oral medicine) 2. The study is divided into two parts in healthy adult volunteers: a single ascending dose (SAD) phase and a multiple ascending dose (MAD) phase, with an additional evaluation of the effect of a high-fat meal on the PK characteristics of FHND1002 3. Enrollment number: 72 4. Designed for ALS
11	A Study on the Safety and Preliminary Efficacy of Aleeto in the Treatment of Amyotrophic Lateral Sclerosis (ALS) Patients	Phase I	NCT06181526	1. Aleeto: This bioactive component originates from stem cell-derived exosomes, representing a collection of microenvironment-specific multiprotein complexes actively secreted through the stress-induced paracrine mechanism in stem cells 2. Intravenous and intrathecal injection 3. Enrollment number: 24
12	An Open-Label, Single-Arm, Single-Center Clinical Study Evaluating the Safety and Tolerability of Regulatory T Cell (Treg) Therapy for Neurodegenerative Diseases	Phase I	NCT06671236	1. Cell Therapy 2. NP001 cell injection are manufactured ex vivo to yield enriched Tregs 3. To evaluate NP001 cell injection at the dose of 10^6^ cells, 10^7^ cells, and 10^8^ cells/times, with up to 3 times separated by 4 weeks among dosing 4. Enrollment number: 12
13	A Study on the Food Effect on the Pharmacokinetics of Oral FB-1071 in Healthy Adult Chinese Subjects	Phase I	ChiTR20230343	1. Chemical medicine (oral medicine) 2. Enrollment number: 15 3. Designed for ALS
14	An Open-Label, Dose-Escalation, Early-Phase Clinical Study Evaluating the Tolerability, Safety, and Efficacy of Intrathecal Administration of VGN-R13 in Patients with Amyotrophic Lateral Sclerosis (ALS)	Phase I	NCT06849609	1. Gene therapy 2. AAV-medicine 3. Intrathecal injection 4. Enrollment number: 6
15	A Study on the Therapeutic Effects of Ciprofloxacin Combined with Celecoxib in the Treatment of Amyotrophic Lateral Sclerosis (ALS)	Investigator Initiated Trial	ChiCTR2500095560	1. Combination and drug repurposing 2. To observe the trend of specific ALSFRS-R score and survival time of ALS patients who have received ciprofloxacin and celecoxib at the same time 3. To observe the changes of blood test indexes (mainly inflammatory factor levels), electromyography results, and lung function in ALS patients 4. Enrollment number: 30
16	A Bridging Study Evaluating the Pharmacokinetics (PK), Safety, and Tolerability of CB03–154 Tablets in Healthy Chinese Subjects	Phase I	NCT05499260	1. CB03–153: a KCNQ2/3 potassium channel activator 2. To assess the safety and tolerability of CB03–154 tablets 3. To assess the impact of oral co-administration of regular meals 4. Enrollment number: 60
17	A Single-Arm, Single-Center, Open-Label Clinical Study Evaluating the Use of HS-03 Injection in the Treatment of Amyotrophic Lateral Sclerosis (ALS)	Investigator Initiated Trial	ChiCTR2400079885	1. Intravenous injection 2. To evaluate the safety and efficacy of HS-03 injection in the treatment of ALS 3. To explore the effects of HS-03 injection on blood inflammatory factors and cells, oxidative stress levels and NfL
18	A Randomized, Double-Blind, Placebo-Controlled, Multicenter Clinical Trial Evaluating the Safety, Efficacy, and Pharmacokinetics of TP04HN106 in Patients with Amyotrophic Lateral Sclerosis (ALS)	Investigator Initiated Trial	ChiCTR2500097214	1. Small molecular medicine 2. Intravenous injection 3. Enrollment number: 60
19	A Clinical Study on the Use of Crizotinib in the Treatment of Amyotrophic Lateral Sclerosis (ALS)	Phase II	ChiCTR2400092197	1. Crizotinib: an endothelin receptor antagonist (Oral medicine) 2. To evaluate the effect of crizotinib on necroptosis and inflammatory markers in ALS patients 3. To evaluate the safety and efficacy of crizotinib in the treatment of ALS 4. Enrollment number: 30
20	A Randomized, Double-Blind, Parallel-Controlled Study on the Effects of Edaravone Dexborneol Injectable Concentrate on Inflammatory Factors in Patients with Amyotrophic Lateral Sclerosis (ALS)	Post-Marketing Study	ChiCTR2400082879	1. An agent composed of edaravone and dexborneol 2. Intravenous injection 3. To evaluate the effect of Edaravone and Dexborneol Concentrated Solution for Injection on the levels of IL-8, TNF-α and il-1β in the peripheral blood of ALS patients. 4. To evaluate the efficacy and safety of Edaravone and Dexborneol Concentrated Solution for Injection in the treatment of ALS. 5. Enrollment number: 30

AAV, Adeno-associated virus; ALSFRS-R, ALS Functional Rating Scale-Revised; ChiCTR, Chinese Clinical Trial Registry identifier prefix; IL-1β, Interleukin-1 Beta; IL-8, Interleukin-8; iPSC, Induced Pluripotent Stem Cell; NCT, ClinicalTrials.gov identifier prefix; NDA, New Drug Application; NfL, Neurofilament Light Chain; No, Number;Phase I/II/III, Trial Designations, Clinical trial phases (I: Safety; II: Efficacy/Dosing; III: Confirmatory); RP2D, Recommended Phase II Dose;. scAAV: Self-complementary Adeno-Associated Virus; TCM, Traditional Chinese Medicine; Th1/Th2/Th17, T-helper cell subsets; TNF-α, Tumor Necrosis Factor-Alpha.Treg, Regulatory T Cells.

## CONCLUSION: NEW ERA OF AMYOTROPHIC LATERAL SCLEROSIS IN CHINA

China's ALS landscape is transitioning towards precision medicine through biomarker-guided diagnostics, genomic integration, and multidisciplinary care models. Social support systems would integrate community-based rehabilitation and digital education to improve quality of life. By synergizing technological breakthroughs, policy optimization, and societal mobilization, China is poised to develop a proactive ALS management framework with global implications for neurodegenerative disease research and care.

The priority is strengthening established frameworks to create a unified national case registry and a dynamic tracking system. Moreover, a multicentre data-sharing mechanism provides an epidemiological foundation for precise prevention and control. Regarding healthcare resource allocation, efforts should be put into building a tiered care network and telemedicine system, emphasising improving primary institutions’ ability to diagnose and treat neurodegenerative diseases, thereby reducing regional disparities in medical standards. In therapeutic translation, clinical research on targeted therapies and gene therapies would be accelerated, with population-specific treatment protocols for Chinese patients validated through international multicentre clinical trials. Concurrently, reforms in the healthcare insurance system should enhance access mechanisms for special medications by dynamically integrating innovative therapies into the national medical insurance catalogue, thereby effectively reducing the financial burden on patients.

## Acknowledgements

*None.*

### Financial support and sponsorship

*Dongsheng Fan was funded by the National Natural Science Foundation of China (81873784 and 82071426) and the Clinical Cohort Construction Program of Peking University Third Hospital (BYSYDL2019002). Ji He was funded by the National Natural Science Foundation of China (82471447), Beijing Natural Science Foundation (L242033), Capital's Funds for Health Improvement and Research (2024-3-3011), and Beijing Physician-Scientist Training Program (BJPSTP-2024-03).*

### Conflicts of interest

*There are no conflicts of interest.*

## REFERENCES AND RECOMMENDED READING

Papers of particular interest, published within the annual period of review, have been highlighted as:

▪ of special interest
▪▪ of outstanding interest
